# Assessing development and climate variability impacts on water resources in the Zambezi River basin: Initial model calibration, uncertainty issues and performance

**DOI:** 10.1016/j.ejrh.2020.100765

**Published:** 2020-12

**Authors:** DA Hughes, S Mantel, F Farinosi

**Affiliations:** a Institute for Water Research (IWR), Rhodes University, Grahamstown, South Africa; b European Commission, Joint Research Centre (JRC), Ispra, VA, Italy

**Keywords:** Zambezi River basin, Hydrological models, Calibration, Model uncertainties

## Abstract

**Study region:**

: The Zambezi River basin, one of the most important water resources in sub-Saharan Africa from both a water supply and hydro-power generation perspective.

**Study focus:**

: Calibration of two hydrological models (Pitman and WEAP) that have been established for 76 sub-basins covering the total basin area of about 1 350 000 km^2^. The longer-term purpose of establishing the models is to facilitate scenario analyses of future conditions related to changes in water use and management as well as climate change.

**New hydrological insights for the region:**

: While there are many (inevitable) uncertainties in the data used, as well as the models and calibrated parameter sets themselves, the results suggest that the models are generally fit for purpose in terms of evaluating future changes. There are, however, some parts of the basin where the reduction of identified uncertainties would lead to improved models and greater confidence in their future use. One of sources of uncertainty relates to the existence of several large wetland areas that have impacts on downstream flows, but are difficult to simulate due to the relatively poor existing understanding of the dynamics of water exchange between the river channels and the wetland storage areas.

## Highlights

• Two hydrological models are established for 76 sub-basins covering the whole of the Zambezi River basin.
• While both models were successfully calibrated in most areas, there remain a number of uncertainties, mostly related to the available data.
• The poor simulations for the Shire sub-basins are associated with uncertainties in the observed flow data, as well as the dynamics of Lake Malawi.
• A further source of uncertainty is related to the interactions of some major tributary river systems with adjacent large wetland areas.

## 1 Introduction

In the preface and introductory chapter to his well-known text on rainfall-runoff modelling, Beven (2001) identifies most of the key issues associated with hydrological modelling. The first is that natural hydrological systems are very complex and largely ‘unknowable’, suggesting that modelling them is almost impossible. The second is that, despite the validity of the first issue, we need models because of the limitations of the available measurement systems and the fact that many areas remain ungauged. The third key issue is that there is a ‘plethora’ of available models and it will always be difficult (or impossible) for any individual to review the full range and make an informed choice of a model for a specific task. The fourth is that while some models are developed for research purposes, ‘the ultimate aim of prediction using models must be to improve decision-making about a (practical) hydrological problem’. Box (1979) is often credited with the first use of the expression ‘all models are wrong but some are useful’, which can be construed as both a criticism, as well as a compliment, of hydrological modelling.

The concept that hydrological models are less than perfect in their ability to reproduce reality is reflected in many studies over the last two to three decades that have focused on the uncertainties in modelling predictions (Beven, 1993, 2005; Pappenberger and Beven, 2006; Ivanović and Freer, 2009; Beven and Alcock, 2012; Hughes, 2016). A great deal of the impetus for investigating uncertainty in hydrological modelling resulted from the IAHS (International Association of Hydrological Science) scientific decade (2003–2012) on PUB (Predictions in Ungauged Basins: Sivapalan et al., 2003; Hrachowitz et al., 2013). While many of the contributions to PUB focused on the science of modelling, the issues of practical modelling applications were not ignored (Hughes et al., 2014a). One of the key issues related to practical modelling is the level of detail included in a model and therefore the size of the parameter space. One argument is that fully distributed, physics-based models are too complex for many practical purposes, especially in large basins and relatively data scarce areas, largely because the data requirements may not be satisfied (Beven, 2001). At the other end of the scale, there have also been arguments for parsimonious models (Perrin et al., 2001; Willems, 2014) that minimise equifinality in the parameter space (Beven, 2006). The perceived advantage of parsimonious models is they are more likely to produce unique solutions when applied using automatic calibration procedures. However, there is an alternative argument (Hughes, 2010) that suggests that explicitly simulating the main catchment scale water balance processes can be an advantage in practical hydrological modelling, even if this leads to a larger parameter space and greater equifinality (Savenije, 2001). This is particularly true when a model is to be used to evaluate future scenarios associated with development or climate change impacts. If the main water balance processes (interception, surface runoff, interflow, groundwater recharge and drainage, evapotranspiration, etc.) are not simulated explicitly and appropriately (Kirchner, 2006), then it might be unrealistic to expect a model to be sensitive to changes in rainfall, temperature (evapotranspiration), water use (e.g. groundwater abstraction impacts on baseflow) and land use (Warburton et al., 2010).

Ultimately, practical modelling becomes a compromise between what might be the best model scientifically, and the one that is appropriately structured, given certain constraints associated with the available forcing data, the information that the user wishes to extract from the model results and the scale of the basin being modelled (Hughes, 2015). These constraints were taken into consideration in selecting the two models that have been used in this study that was designed to establish appropriate hydrological and water resources development modelling tools for the whole of the Zambezi River basin (approximately 1 350 000 km^2^ in area). This paper briefly presents the models, the results of establishing (calibration and validation) the models to represent historical conditions, as well as identifying the key modelling uncertainties (related to the models or the data) in the different parts of the basin. A dedicated paper (Hughes and Farinosi, 2020) presents the details of several future scenarios (based on likely/possible future changes in water resources development and climate), how these are incorporated into the models, and the simulated consequences of these future scenarios.

## 2 The Zambezi River basin

**Fig. 1:**
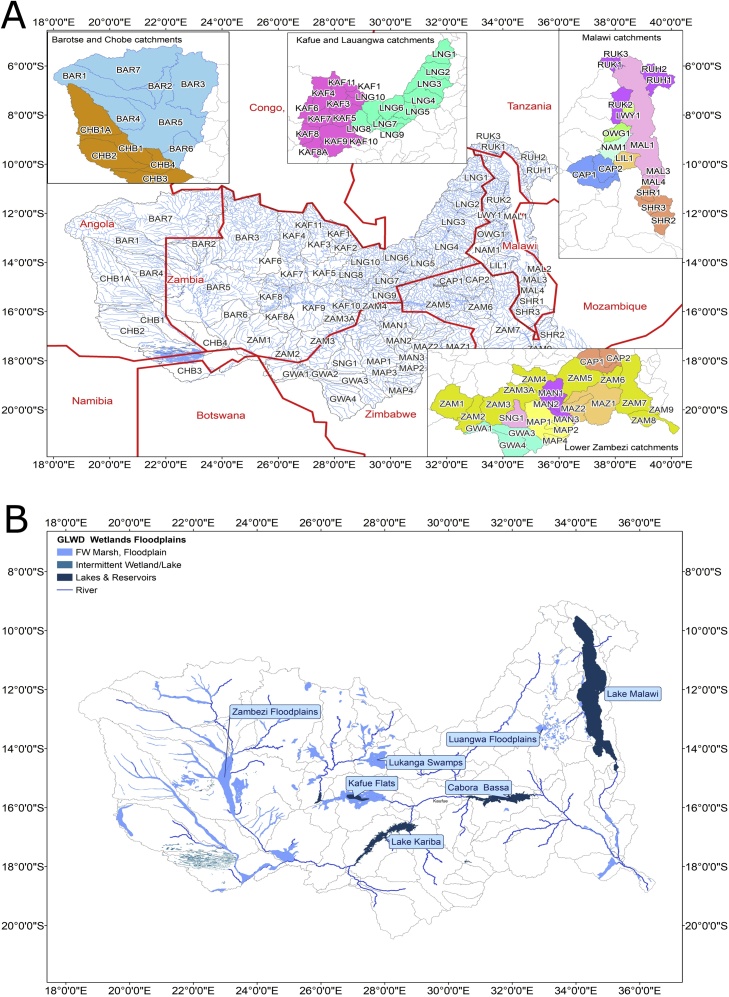
The Zambezi River basin showing the main tributaries, sub-basin division (a) and large wetlands and reservoirs (b).

The Zambezi River is one of the largest rivers in Africa with a total catchment area of about 1 350 000 km^2^, and drains parts of eight countries (Angola, Botswana, Malawi, Mozambique, Namibia, Tanzania, Zambia and Zimbabwe). Apart from the main river, the total basin consists of several major tributary systems including the Luangwa, Kafue, Chobe and Shire rivers (Fig. 1a). Fig. 1b illustrates that there are several major wetland systems (Luangwa and Barotse floodplains, Lukanga swamps, Kafue flats), many more localised wetland systems on the Chobe and Shire rivers, as well as one of the largest natural lakes in Africa (Lake Malawi/Niassa). Mean annual rainfall varies from over 1 200 mm y^−1^ in some of the headwater areas of the Shire and Kafue sub-basins, to less than 700 mm y^−1^ in the more arid parts of Zimbabwe. The rainfall is also highly seasonal, the main wet season being from November to March. Mean annual potential evapotranspiration varies from about 1 200 to 1 700 mm y^−1^, reflecting a similar spatial pattern as the rainfall (higher evapotranspiration in the lower rainfall areas). The majority of the basin is underlain by hard rocks (metamorphic, igneous and sedimentary), while the western areas of the Chobe and Upper Zambezi are underlain by the deep unconsolidated deposits of the Kalahari sands.

The major water resource development impacts are related to the hydro-power dams (including evaporation losses) on the Kafue River (Itezhi-Tezhi and Kafue Gorge), as well as on the main river at Kariba and Cahora Bassa dams (Spalding-Fecher et al., 2017). There are also known (but largely unquantified at the sub-basin scale) localised abstractions for irrigation, mining and domestic use (SADC, 2008). These are not considered to be currently large enough to substantially impact the natural flow regime of the main tributaries and therefore the historical hydrology of the basin upstream of the major dams can be considered to be stationary (Winsemius et al., 2006).

For the purposes of this study, the total basin area has been divided into 76 sub-basins (Fig. 1a and Table 1) that reflect the location of either gauging stations (see table in the supplementary material), key natural features (e.g. wetlands), or existing major water resource development infrastructure (mostly hydro-power reservoirs). The average area of the sub-basins is about 18 000 km^2^, the smallest is 586 km^2^ and the largest 78 252 km^2^. There are 38 sub-basins that have some stream flow or water storage data that are considered potentially useful for model calibration, while a further 8 sub-basins have observed data that are considered too uncertain to be useful. The latter were identified because their data were either inconsistent with other gauged data, or internally inconsistent (large scale changes within the record), or contain too much missing data. While the main river and many of the major tributary systems are gauged, the basin remains a relatively data scarce region in terms of the length and quality of some of the stream flow records, as well as the additional climate and water use data that are required to establish a hydrological model.

**Table 1:** Sub-basins used within the setups of the two models (ungauged includes those areas with gauge data that are considered unreliable).

Sub-system	Type	Identification code (see Figure 1)
Luangwa	Ungauged	LNG1, 2, 3, 4, 6, 8, 9 & 10
	Gauged	LNG5 & 7
	Wetlands	LNG2, 3 & 4
Kafue	Ungauged	KAF6, 8, 9
	Gauged	KAF1, 2, 3, 4, 5, 7, 8A, 10 & 11
	Wetlands	KAF5 & 9
	Reservoirs	KAF8 & 10
Upper Zambezi	Ungauged	BAR1, 2, 5 & 7
	Gauged	BAR3, 4 & 6
	Wetlands	BAR5
Chobe	Ungauged	CHB1, 1A, 2 & 3
	Gauged	CHB4
	Wetlands	CHB1 & 3 (but sub-basins could be included)
Central basin tributaries	Ungauged	GWA1, SNG1, MAP1, MAN1, CAP1 & 2, MAZ2.
	Gauged	GWA2, 3 & 4, MAP2, 3 & 4, MAN 2 & 3, MAZ1
Lake Malawi and Shire	Ungauged	RUK3, OWG1, LIL1, MAL2, SHR2
	Gauged	RUK1 & 2, RUH1 & 2, LWY1, NAM1, MAL1, 3 & 4, SHR1 & 3.
	Wetlands	MAL1, SHR2 & 3.
	Reservoir	MAL2 (for outflow controls from L. Malawi).
Main river	Ungauged	ZAM3A, 4, 5, 7 & 8
	Gauged	ZAM1, 2, 3, 6 & 9.
	Reservoirs	ZAM3 & 5

## 3 Models and calibration methods

While other models have been previously applied in some parts of the basin (Winsemius et al., 2006; Kling et al., 2014; Jury, 2017), the two models used in this study are the Water Evaluation And Planning (WEAP) model (https://www.weap21.org/: accessed November 2019) and the Rhodes University version of the Pitman model (Pitman, 1973; Hughes, 2013). Both are semi-distributed conceptual type models that operate at a monthly time step and both have been previously applied in parts of the Zambezi River basin, as well as other areas of sub-Saharan Africa (Cervigni et al., 2015; Hughes, 2016; Spalding-Fecher et al., 2017; Hughes and Mazibuko, 2018). The choice of these models was based on the authors existing calibration experience, the frequency of use of the models in sub-Saharan Africa and because both represent compromises between detailed and simple models, and either explicitly or implicitly include the key runoff generation processes expected to occur within the Zambezi. Both models can represent a range of development impacts, albeit in different ways, both are packaged within application systems that include a spatial interface (map of sub-areas, nodes and other information) and tools for data management and for reviewing the simulation results. More details can be found about the WEAP model at https://www.weap21.org/, while further details on the Pitman model can be found by downloading (https://www.ru.ac.za/iwr/research/spatsim/) the SPATSIM (SPatial And Time Series Information Modelling) software, which is a generic modelling framework within which the IWR version of the Pitman model (as well as other models) is applied (Hughes and Forsyth, 2006). The available support material for both models contain extensive lists of model applications documented in published papers, scientific reports or postgraduate theses. All of the evidence therefore suggests that both of these models are fit for purpose in terms of practical hydrological and water resources simulation modelling in the Zambezi River basin.

In general terms, the WEAP model is simpler with fewer parameters than the Pitman model, however, it is also quite flexible and users can (to a certain extent) modify the default algorithms to create a different structure. Previous experience of applying both models to the same basins suggests that it is possible to align the WEAP algorithms to approximately match the outputs from the Pitman model (Cervigni et al., 2015), but also that there are some key differences in some situations. The first is that the ground water approach in the Pitman model (Hughes, 2004) is more complex than the WEAP model. The total recharge leaving the main moisture storage zone of the Pitman model can be subject to evapotranspiration in the riparian channel margins, before contributing to stream flow. The Pitman model can therefore simulate alternating periods of ground water contributions and periods of no contribution (Hughes, 2004). While the WEAP model can simulate variable volumes of ground water outflow, it cannot simulate periods with and without outflows, unless the default algorithms are modified to include an upper zone moisture store threshold, below which there is no outflow. Even then, the model always simulates some flow even if it is very small. There are also differences in the approaches to the simulation of recharge and interflow processes. The WEAP model uses a single upper zone conductivity parameter and a fixed (a value of 2.0) power relationship with relative upper zone moisture content. The total outflow is then partitioned between interflow and ground water recharge. The Pitman model uses separate non-linear functions for these two processes, each with maximum outflow and power parameters.

A further key difference between the models in the context of the Zambezi River basin is that the Pitman model includes an explicit sub-model to simulate the exchange processes between river channels and large wetlands (Hughes et al., 2014b). The approach adopted in the WEAP model is to create a reservoir with release operating rules to represent the outflows from wetland storage, as well as a bypass channel to divert flows from upstream that are assumed to pass through the wetland. While this makes it quite difficult to quantify the various components of the reservoir/bypass system in the WEAP model, it is also acknowledged that the wetland sub-model of the Pitman model is not simple to parameterise without detailed wetland information (Makungu, 2019). The Pitman model also has a channel routing function that can be used to delay and attenuate upstream flows through large sub-basins, or basins with large and low gradient channels (Hughes et al., 2006). No such function exists in the WEAP model.

With respect to development impacts, both models are able to represent direct abstractions, as well as reservoir storage, abstractions and downstream releases. Arguably, the WEAP model has more facilities to represent water resources operating rules (supply curtailments and priority rules for competing demands) than the Pitman model. The Pitman model includes a simple function to represent aggregated farm dam storage and abstractions within a sub-basin (using a parameter representing the % of the area supplying the dams). A separate sub-basin node would have to be created to achieve the same objective in the WEAP model.

### 3.1 Forcing data

Both models were forced using the University of East Anglia, Climate Research Unit data (https://crudata.uea.ac.uk/∼timm/grid/CRU_TS_2_1.html, accessed during Oct. 2019) which is available from 1901 to 2017 at a grid scale of 0.5° (Harris et al., 2014). The potential evaporation data for the Pitman model are based on the LISVAP calculations (Alfieri et al., 2019) based on the ERA5 data for 1979–2018 (https://confluence.ecmwf.int/display/CKB/ERA5+data+documentation, accessed during Oct. 2019). The reference evapotranspiration inputs to the WEAP model are based on historical gridded (0.25 degrees) time-series climate data (temperature, precipitation and wind speed) for 1948–2010 generated by the Terrestrial Hydrology Research Group at Princeton University (Department of Civil and Environmental Engineering/Princeton University, 2006; Sheffield et al., 2006). Actual evapotranspiration in WEAP is calculated using the Rainfall-Runoff Soil Moisture method (https://www.weap21.org/WebHelp/Two-bucket_Method.htm) which utilises the potential evapotranspiration, crop coefficient factor and the upper and lower zone soil moisture states in a two-bucket model.

### 3.2 Calibration methods

Both models are traditionally calibrated using manual parameter adjustments to obtain good representations of the available observed data, coupled with relatively subjective parameter transfer approaches to un-gauged areas based on sub-basin physical and climate similarity. This can be an effective approach when there are observed data available at several points (headwater and downstream) in the modelling system. The assessment of alternative parameter sets is guided by visual comparison of time series, flow duration curves (FDCs) and seasonal distributions and backed up within the SPATSIM system with the calculation of several objective functions. These include the % bias in mean monthly simulated volume and the Nash coefficient of efficiency (CE), using both untransformed and natural log transformed flows. The WEAP model simulations can be exported and imported into SPATSIM to calculate the same objective function values.

While automatic calibration approaches are often favoured, they are not always very successful in regions where there are potential problems with the rainfall forcing data and when the models being calibrated contain a relatively high level of equifinality (which both models do). Part of the problem is that the automatic calibrations can generate spurious parameter sets as they try to fit the model to parts of the time series where the rainfall data might be inadequate. If these can be identified, a manual calibration process can ignore these periods. However, the IWR version of the Pitman model also includes an option to use a simple random parameter sampling scheme (using a uniform distribution), using plausible ranges of some (or all) parameters to generate ensemble outputs. These outputs can be used to more rapidly explore the model domain for groups of parameter values that generate the best results compared with all or parts of the observed data time series. They can also be used to identify different parameter combinations that produce broadly similar results (equifinality). For example, in some situations the two surface runoff functions of the Pitman model (Table 2) may produce equally acceptable results, while the low flow regime may be equally well simulated with either interflow or ground water outflow as the dominant process. Without further site specific information about real processes it may not be possible to resolve such equifinalities, but it is useful to know that they exist. This version of the model has been previously applied in several areas of the basin (Hughes, 2013; Hughes and Mazibuko, 2018) and the results were very useful in guiding the manual calibration process. Several additional runs were made during the current calibration study to further explore the parameter space for parameter sets that yield satisfactory model performance. While a similar facility is not available for the WEAP model, the Pitman model results can still be useful to guide the manual calibration of the WEAP parameters.

**Table 2:** Summary of simulation results for both models using the four objective function statistics.

Sub-basin	Data period	Statistics: % bias : CE : %bias(ln) : CE(ln)							
		Pitman				WEAP			
LNG5	1980−1989	−2.2	0.877	0.5	0.932	−0.1	0.731	−4.5	0.828
LNG7	1950−2014	−3.9	0.714	2.0	0.742	−9.2	0.536	2.7	0.626
KAF11	1959−2005	−5.5	0.666	1.5	0.775	−9.7	0.199	2.0	0.684
KAF1	1952−1986	−1.8	0.877	2.3	0.862	−4.9	0.545	−0.3	0.789
KAF2	1963−1998	−4.6	0.839	0.6	0.870	−1.3	0.477	−1.1	0.785
KAF3	1969−1992	−3.8	0.785	2.3	0.832	−2.8	0.516	1.4	0.745
KAF4	1959−2005	−1.8	0.733	−0.7	0.704	0.2	0.656	0.0	0.758
KAF5	1953−1992	−5.3	0.812	1.0	0.816	−4.0	0.819	1.7	0.780
KAF7	1973−2016	−5.0	0.650	1.1	0.802	−7.3	0.633	1.8	0.799
KAF10	1974−2010	−7.5	0.584	1.1	0.492	−11.5	0.295	−0.9	0.204
BAR3	1959−2012	−2.4	0.689	0.9	0.736	3.8	0.700	2.1	0.764
BAR4	1961−2013	0.2	0.644	0.9	0.703	−3.6	0.399	2.4	0.377
BAR6	1943−2017	−0.3	0.775	1.8	0.842	−4.5	0.692	3.1	0.728
ZAM1	1924−2007	9.8	0.643	1.7	0.773	15.3	0.345	4.6	0.503
ZAM2	1927−2007	8.3	0.784	1.1	0.834	14.3	0.676	4.5	0.662
GWA2	1955−1996	−7.5	0.537	−4.1	0.399	10.5	0.695	−39.8	0.113
MAP2	1950−2017	2.3	0.502	−0.3	0.011	−6.5	0.488	19.6	0.208
MAP3	1957−2016	2.9	0.416	110	0.044	2.1	0.501	−105	−0.142
MAP4	1955−2017	2.0	0.396	−16.4	0.006	−5.9	0.398	−200	−2.413
MAN3	1965−2017	−3.7	0.636	−43.5	0.733	9.0	0.258	−107	−4.244
MAN2	1993−2017	−6.4	0.461	−21.1	0.200	−4.5	0.311	−83.5	−1.192
MAZ2	2003−2017	−1.6	0.515	−5.8	0.322	−3.5	0.125	−16.2	−0.008
RUH2	1971−2017	−5.6	0.654	−1.0	0.693	−2.0	−1.813	−8.8	−1.653
RUH1	1990−2007	−4.8	0.808	−0.4	0.798	−19.1	0.539	−3.2	0.698
RUK1	1985−1997	−1.6	0.654	−1.0	0.702	−37.9	0.301	−13.5	0.101
RUK2	1986−2008	4.1	0.736	0.4	0.837	−9.5	0.467	−3.5	0.812
LWY1	1953−1994	3.1	0.529	1.7	0.686	−6.7	0.386	−2.8	0.603
NAM1	1957−2000	−1.9	0.720	7.3	0.691	−6.0	0.479	17.8	0.532
ZAM3	1961−2007	−2.5	0.309	0.1	0.233	−3.1	0.285	0.4	0.331
ZAM6	1951−2004	−0.1	0.519	−0.2	0.355	−0.5	0.427	−0.9	0.246

Split sampling verification has not been used in this study, partly because some of the observed records are quite short, meaning that the samples used would be even shorter. A further reason is the rainfall inputs are known to be uncertain based on previous experiences of using these data in the region (Hughes et al., 2011a), but we do not know which parts of the record might be more uncertain than others and this could bias a split sample testing approach. The key component of validation in this study is therefore that good calibration results for upstream sub-basins should also result in good calibration results for downstream sub-basins (Hughes et al., 2006). The main objectives of the study are not to scientifically prove that the models are applicable (this has been done before in this region with both models), but to determine where (and where not) acceptable simulations can be achieved that are useful for future assessments of water resources availability and management. This includes identifying the key uncertainties in both the models and the available data (and the spatial variability of such uncertainties) so that these can be targeted in future studies or data collection programmes.

## 4 Calibration results

Table 2 presents the data periods used for comparison and the four objective function statistics that are used to summarise the model performance for the two models and for all the sub-basins with useful observed data. The following paragraphs discuss the model performance and key uncertainty issues for each of the major sub-systems of the Zambezi River basin (Fig. 1a). Some qualitative assessments of the model results in different parts of the basin are included in the text using words such as ‘reasonable’, ‘quite poor’, ‘very bad’, etc., These evaluations are based on several criteria that include the four main objective functions applied to the time series data, but also include assessments of the correspondence of the simulated FDC and seasonal distributions to the observed data. ‘Reasonable’ or better could be defined as Nash Sutcliffe efficiency values above 0.5 and bias values of less than ± 10 %, coupled with similar FDCs and seasonal distributions to the observed.

### 4.1 Luangwa River

A key geomorphological feature of the western parts of the Luangwa River basin is the presence of Dambos, which are relatively small, but frequent, valley bottom wetlands that have the potential to generate saturated surface runoff during the early part of the wet season (Hughes and Mazibuko, 2018). The other key feature is the Luangwa River floodplain, although recent work suggests that the downstream impacts (attenuation and delay) are quite minimal at the monthly time scale (Makungu, 2019) and no wetland effects were included in the WEAP model. While both models produce acceptable results at LNG5, the slightly better results for the Pitman model might reflect the inclusion of the wetland sub-model. The main difference between the results at the downstream gauge (LNG7) is that the WEAP model tends to predict a wet season that peaks approximately one month too soon.

### 4.2 Kafue River

The Kafue River is well gauged, but it is also quite complex with two major wetlands, the Lukanga swamps (KAF5; Mwelwa, 2004) and the Kafue Flats (KAF9). The Itezhi-Tezhi dam (KAF8), located upstream of the Kafue flats, is used for controlled releases to the Kafue Gorge hydro-power dam (KAF10). The pattern of controlled releases used within the models was based on some observed data for part of the modelling period. There are known to be some abstractions along most of the length of the main channel, but these are poorly quantified. The results for the Pitman model down to the gauge at Hook Bridge (KAF7) are encouraging, and reasonable results were obtained below Kafue Gorge dam (KAF10), despite the complications of trying to capture the release patterns from the two reservoirs as well as the effects of the Kafue Flats wetland. The WEAP results for some of the upstream sub-basins are relatively poor and the main differences between the two models are that the wet season peaks too soon in the WEAP simulations, while the WEAP FDCs are sometimes better than the Pitman model.

### 4.3 Upper Zambezi and Chobe River

This sub-system is arguably the most complex of the western half of the Zambezi River basin, partly because of the Kalahari sands in the extreme western parts, several major wetlands (including the Barotse floodplain), and partly because of some confusing signals from some of the available observed data. The observed records suggest that the main eastern tributary (Kabompo River at BAR3) has a quite steep FDC, which is further reflected in the data downstream below Ngonye Falls (BAR5 and BAR6). However, the observed records for the other main gauged tributary (BAR7) suggest very high baseflow contributions that are incompatible with the flow records further downstream below Ngonye Falls (BAR6) and at Victoria Falls (ZAM1). These gauging data therefore need to be further examined in the future to try and correctly resolve this source of uncertainty.

**Fig. 2:**
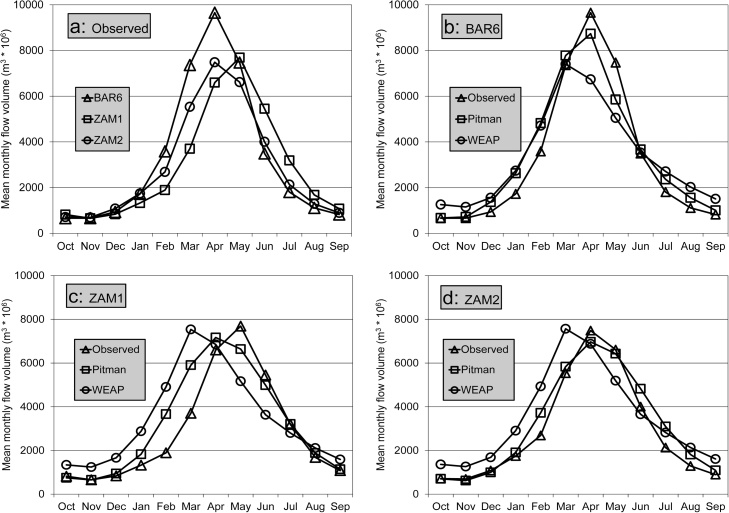
Seasonal distributions of observed (a) and simulated flow volumes for the Upper Zambezi at Katima Mulilo (b: BAR6), Victoria Falls (c: ZAM1) and above Kariba Dam (d: ZAM2). ZAM1 and ZAM2 have lower flows as the records are longer and more complete than BAR6.

Table 2 suggests that the simulations for both models are less successful than in the previous two systems, but that the results remain reasonably satisfactory given the uncertainties associated with the data and the effects of the Barotse floodplain. The relatively poorer results for the WEAP model below Ngonye Falls (BAR6), and further downstream (Fig. 2), are partly related to the difficulties of representing non-linearities in the channel-wetland exchange processes compared to the Pitman model. Neither of the models were able to adequately simulate the patterns of observed flows for the Chobe sub-system, which is relatively flat and underlain by Kalahari sands. While including wetlands in the Pitman model helped to a certain extent, the CE values remained below zero. However, the overall contribution of the Chobe River to the Zambezi River is very small.

Both of the main Zambezi River gauges (ZAM1 and 2) above Kariba Dam have quite long records, with few missing data, and are generally consistent with each other. However, the peak season recorded flows at ZAM2 occur, on average 1 month before those at ZAM1 (Fig. 2a), which clearly points to some data problems. The problem seems to lie mostly with the gauge at Victoria Falls (ZAM1), as the other two gauges (BAR6 and ZAM2) have similar seasonal distributions (Fig. 2a). The focus of the calibration effort was therefore on BAR6 and ZAM2, at the expense of ZAM1.

### 4.4 Central basin tributaries

Most of the central basin tributaries drain the semi-arid areas of Zimbabwe and have either seasonal or ephemeral flow regimes with quite substantial periods of zero flow. However, the length of zero flow periods in the gauged record (compared to natural conditions) could be affected by upstream abstractions (longer), or reservoir releases and return flows (shorter). These types of catchments are difficult to simulate with coarse spatial and temporal scale models as they are generally not able to fully capture the high degree of variability in both rainfall and runoff response. The main focus of the calibration exercise was therefore to simulate the FDC characteristics as well as possible.

**Fig. 3:**
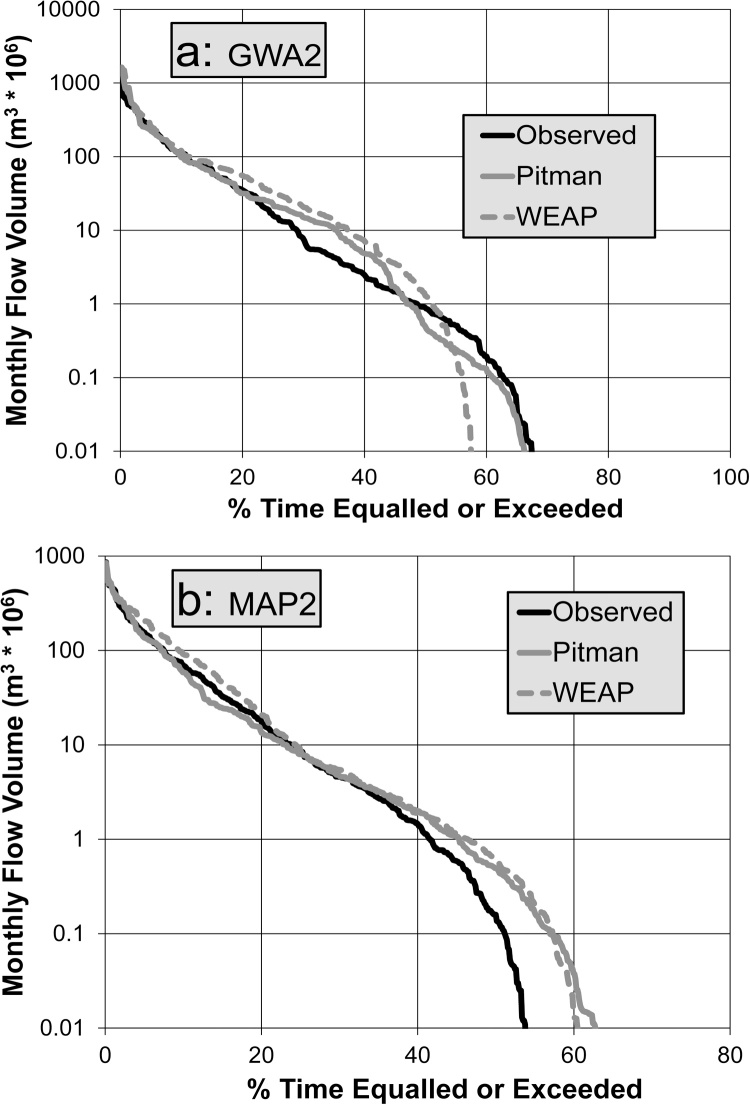
Flow duration curves for observed and simulated (Pitman and WEAP) monthly flow volumes for sub-basins GWA2(a) and MAP2(b).

The Gwayi River sub-system is gauged at four points, but the downstream gauge has a short record (1996 to 2014: Supplementary table) and a very high proportion of missing data. The results for the two main headwater sub-areas are quite poor, while the simulations improve somewhat at the Mamatavi gauge downstream (GWA2: Table 2 and Fig. 3a). The main parts of the catchment are highly cultivated but there is no evidence of any large dams or extensive irrigation. The Senyati River (MAP2, 3 and 4), Manyame River (MAN2 and 3) and Mazoe River (MAZ1 and 2) sub-systems are quite heavily utilised for agriculture, supported by both large reservoirs and many smaller farm dams. However, the storage and demand information is highly uncertain and the water use could be highly non-stationary. Table 2 and Fig. 3b (MAP2: Umfuli River) indicate that the results are similar to site on the Gwayi (GWA2), with quite poor statistics, despite reproducing the upper ends of the observed FDCs very well. The downstream Mazoe data at Luenha (MAZ1) are inconsistent with the upstream data and were not used in the calibration. The final tributary in this sub-region is the Capoche River (CAP1) joining the Zambezi River immediately downstream of Cahora Bassa Dam. Unfortunately, the gauging records are too uncertain to be useful for model calibration.

In some sub-basins, the models do not simulate the low flows and periods of zero flow very well. To achieve zero flows for the WEAP model, it was necessary to use a conditional expression with a storage threshold to limit interflow and groundwater outflow, to replace the fixed conductivity parameter values. In the Pitman model, groundwater outflow can be limited by using the riparian strip evapotranspiration loss parameter. Part of the difficulty with simulating the low flow regimes of these rivers is also likely to be due to non-stationary water uses, as well as the possibility of artificial downstream releases from the larger reservoirs (e.g. within MAP3, MAP4 and MAN3).

### 4.5 Lake Malawi/Nyasa sub-system

There are six gauged tributaries flowing into Lake Malawi/Nyasa, while data for two additional gauges (RUK3 and LIL1) have been rejected due to excessive runoff coefficients (66 % and 69 %, respectively), compared to other catchments in the same area (<20 %). Comparisons of the CRU rainfall data with an alternative data set from the University of Delaware (UNIDEL; Willmott and Matsuura, 2001), reveals that the UNIDEL data are 18 % and 69 % higher than the CRU data for RUH2 and RUK3, respectively, suggesting that the CRU data are not suitable for some parts of the Lake Malawi sub-system. There are also some inconsistencies within some of the gauged records, such that some periods were rejected for calibration purposes. The results for the downstream gauge on the Ruhuru River (RUH1), the Songwe (RUK1), the South Rukuru (RUK2) and the Bua (NAM1) rivers are considered acceptable (Table 2), further evidenced by good reproductions of the observed FDCs and seasonal distributions. Overall, the Pitman model performed better than the WEAP model in these sub-basins (Table 2).

**Fig. 4:**
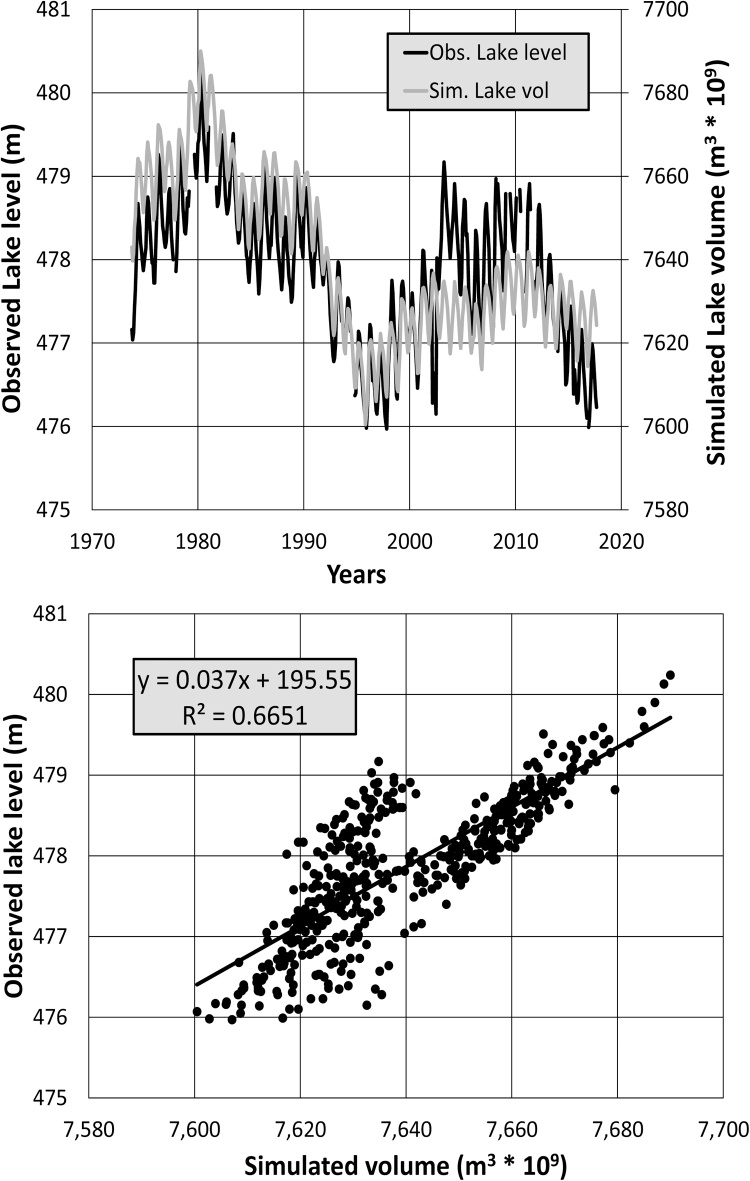
Time series (1973 to 2017) comparisons of observed Lake Malawi/Nyasa levels and simulated volumes using the Pitman model, and the regression relationship between these two variables.

For the Pitman model, the wetland sub-model was used to try and simulate the dynamics of Lake Malawi and to calibrate the outflows against the observed flows at the outlet (MAL1). In the WEAP model the lake impacts are simulated using a reservoir with bypass flows. However, the calibration of both models is severely affected by the uncertainties in the quality of the data at MAL1. There are five gauges on the Shire River downstream of the lake, but they are highly inconsistent with each other, not only in terms of the variability of monthly flow volumes, but also in terms of the seasonality. For example, the observed flows for MAL1 peak in August, for MAL3 and MAL4 in May, and for SHR1 and SHR3 in March. The simulated outflows peak in April/May, as much as 3 months earlier than the observed outflows. Not surprisingly, the Nash coefficients are very poor (≤ 0.35), despite the volume bias statistics being relatively low (≤ ±5%). The results further downstream along the Shire River are even worse with very low or negative Nash coefficients, partly related to the lack of reliable observed flow data signals, inadequate simulations upstream, and the wetland dynamics in parts of the Shire River. These results clearly point towards the need for much more information about the hydrological dynamics of this part of the Zambezi River basin. However, the simulated lake volumes compare quite favourably to some observed water level data (1973–2017) at Mbamba Bay in Tanzania (Fig. 4), with a regression coefficient of about 0.66 despite a period with poor correlations between 2002 and 2012 (for 1973–2001, the regression coefficient increases to 0.92). The observed and simulated water level seasonal distributions are also close, both peaking in April/May.

### 4.6 Main Zambezi River, including Kariba and Cahora Bassa dams

The operation of the two dams has not been very consistent over the period of historical records, and they were simulated using the available information on controlled releases and spills. Gandolfi and Salewicz (1990) indicate that Kariba dam is partly operated for flood control and that managed releases are made early in some wet seasons to prevent the reservoir from spilling. The downstream observed record was used to try and establish a pattern of flood releases (dependent upon reservoir storage volume) that improved the simulations.

**Fig. 5:**
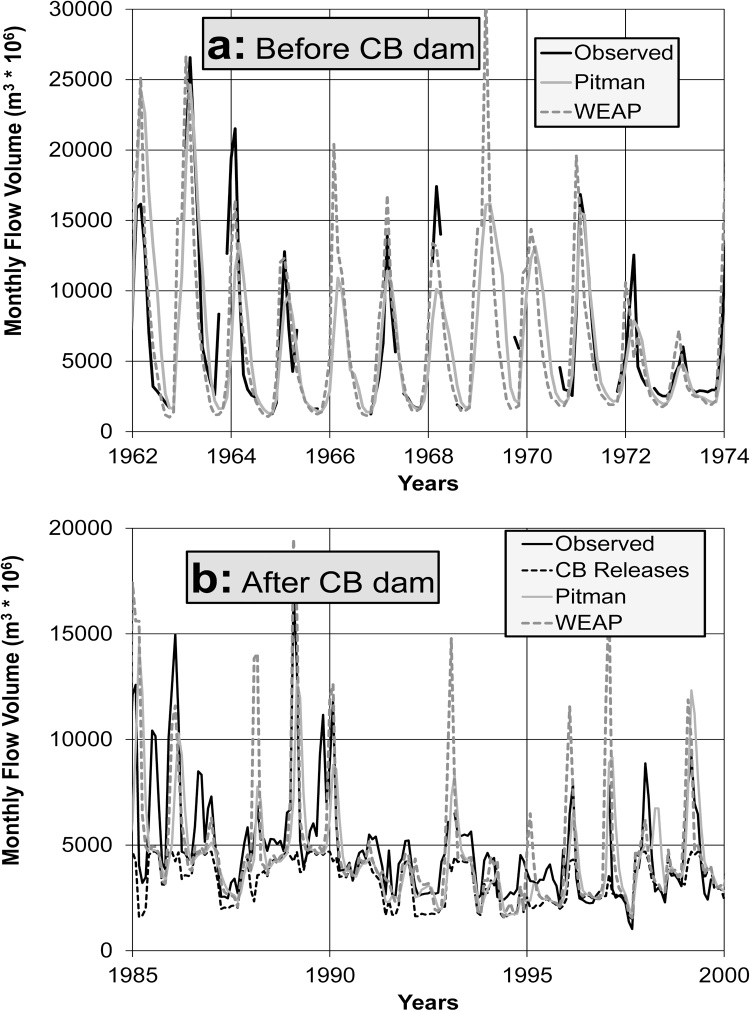
Time series comparisons of observed and simulated flow for ZAM6 at Tete in Mozambique for periods before (a) and after (b) the construction of Cahora Bassa (CB) dam.

Although there are some gauge data at ZAM3A (just above the confluence with the Kafue River), the available data appear to be highly suspect. Gauge data exist at Tete (ZAM6) and ZAM9 (below the Shire River confluence), but the latter is of little value for calibration due to the dominant influence of the poorly simulated Shire River sub-system. The statistical comparisons of Kariba (ZAM3) outflow volume are relatively poor, but improve downstream at Tete (Table 2 and Fig. 5). The statistics given in Table 2 are based mostly on the longer post-dam period (Fig. 5b), and are inevitably affected by the uncertainties in the flood control operations of both dams. In general terms, both models produce quite similar results at Tete (ZAM6) with the Pitman model being slightly better in terms of FDC and seasonal distribution patterns.

## 5 Model validity with respect to simulated hydrological processes

A key issue associated with the scientific and practical value of hydrological model results is whether an acceptable calibration has been achieved for the right reason (Kirchner, 2006). If not, the model may not be sensitive to at least some future changes in either climate or land use. However, it is impossible to validate a model in terms of the relative contributions of the components of evaporative losses and runoff generation, because we do not, and probably never will, know what these are at the coarse scale of the sub-basins used for the Zambezi River basin. The alternative is to suggest that if we get results that satisfy a wide variety of fitting criteria, then we can assume that we are acceptably close in simulating the results for the right reason. These criteria include, not only the overall fit statistics based on the time series of monthly flow (Table 2), but also the shapes of the seasonal distributions and flow duration curves. Similarly, getting appropriate FDC shapes for semi-arid catchments (including the length of time of zero flows) cannot really be achieved without getting the right balance (at the catchment scale) between surface runoff (dominant) and intermittent interflow or groundwater inputs to the channel.

**Fig. 6:**
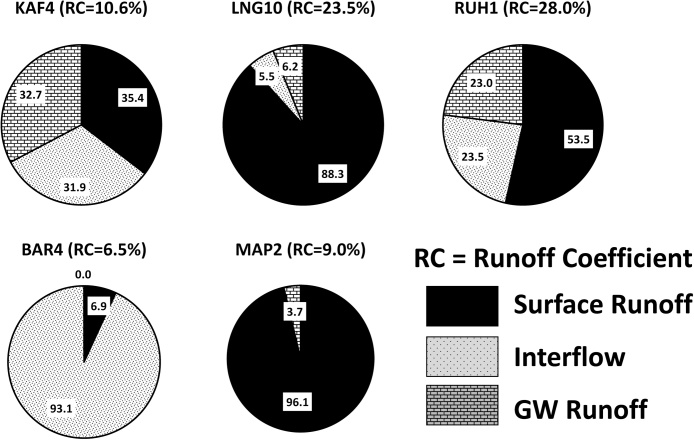
Simulated runoff components for selected sub-basins.

Fig. 6 shows the total runoff partitioning into surface runoff, interflow and groundwater outflow to the channel for selected sub-basins using the Pitman model results. The WEAP model results are very similar as the parameterisation was guided by the parameter sets used for the Pitman model. In general terms, the partitioning shown is consistent with what we expect in the different parts of the Zambezi, as well as with previously reported results for similar areas (Winsemius et al., 2006; Hughes and Mazibuko, 2018). KAF4 on the Lunga River is quite typical of many of the sub-basins of the Kafue and Luangwa river systems, where there is an almost equal split between the three runoff components and the surface runoff is dominated by saturation-excess runoff due to the presence of Dambos. The two major western tributaries of the Luangwa River (LNG8 and LNG10) required high saturation-excess surface runoff to get the correct seasonal distribution at the downstream gauge (LNG7). RUH1 represents typical partitioning in the gauged tributaries of Lake Malawi and is quite similar to the sub-humid sub-basins of the Kafue.

The results for the Luanginga River at BAR4 are not very good (Table 2) but Fig. 6 illustrates the partitioning that might be expected for some of the areas in the west of the Upper Zambezi underlain by Kalahari sands, resulting in a low proportion of surface runoff and either high interflow or groundwater contributions. It is quite difficult to resolve whether interflow or groundwater should dominate without more information about the real processes. MAP2 represents a typical partitioning for the semi-arid sub-basins where the dominant runoff process is assumed to be infiltration excess surface runoff with some intermittent groundwater contributions during more prolonged wet periods.

## 6 Discussion and conclusions

**Fig. 7:**
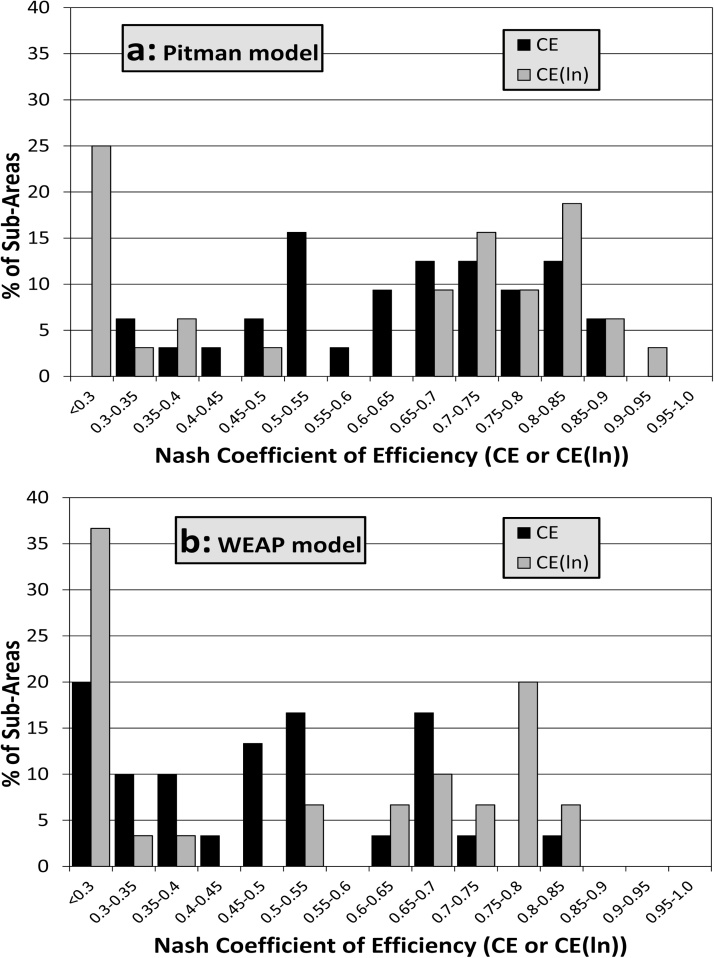
Frequency distributions of simulation results using the Nash coefficients of efficiency for both models and all sub-basins listed in Table 3.

For a large part of the Zambezi River basin the Pitman model results, and to a somewhat lesser degree, the WEAP model results, are considered to be acceptable and fit for purpose, given the large number of uncertainties involved in this study (Table 2 and Fig. 7). The main focus of this discussion is to summarise these uncertainties and attempt to identify those that might potentially be reduced, given realistic expectations about the availability of additional data, and/or further efforts to improve the model calibrations.

It is immediately apparent from Table 2 and Fig. 7 that the Pitman model has produced better results overall than the WEAP model. Part of this conclusion is related to the greater experience of the authors in the application of the Pitman model within the southern Africa region, given that both models were largely calibrated using subjective manual adjustment of the parameter values. Whether a more experienced WEAP model user could achieve appreciably better results (possibly using the expression builder to modify the default model structure) is a moot point. However, the tools available within SPATSIM to facilitate the calibration of the Pitman play a major role in achieving improved calibration. This includes the parameter exploration version of the model that allows ensemble outputs to be generated based on sampling from a priori parameter bounds. One of the recommendations of this study is that improved calibration tools could be added to the WEAP model interface. Following one of the review suggestions, it would also be useful to add the popular Kling-Gupta-Efficiency criteria (Gupta et al., 2009) to the standard objective functions calculated within the SPATSIM interface for the Pitman model.

This paper has not presented any uncertainty analyses of the model results, partly because the objective was to cover the whole basin and space does not permit a lengthy discussion about uncertainty analysis, and partly because uncertainty analyses have not been completed for all the sub-basins. However, it would be a relatively straightforward task to implement a full uncertainty analysis, using the Pitman model, now that we have some initial calibrations and a reasonably acceptable understanding of the runoff characteristics of all the sub-basins, based on validations using downstream gauging stations. The major uncertainties from a practical water resources perspective across most of the basin, lie in the projections of future water availability, the subject of the follow on to this paper (Hughes and Farinosi, 2020). If water resources management decisions need to be taken in the largely ungauged parts of the basin, then it will be beneficial in the future to implement a more thorough uncertainty analysis of the historical flow simulations.

A number of decisions were taken about the quality of the observed data and whether to reject some records and keep others. These decisions were largely subjective as we did not have access to the raw data (stage records and stage-discharge relationships). While some of the decisions are considered to be quite robust, for example those based on excessive runoff coefficients, others need to be further checked to ensure that both useful data have not been unnecessarily rejected, and that poor data have not been used for calibration. A key recommendation is that a dedicated assessment of all the observed stream flow and reservoir storage data should be initiated. This assessment will clearly require the cooperation of the national hydrometric agencies of all the riparian countries, but the benefits should be self-evident. The outcomes will help to remove some of the uncertainties in the model setups, as well as provide possible guidelines for future hydrometric observation activities within the basin as a whole.

**Fig. 8:**
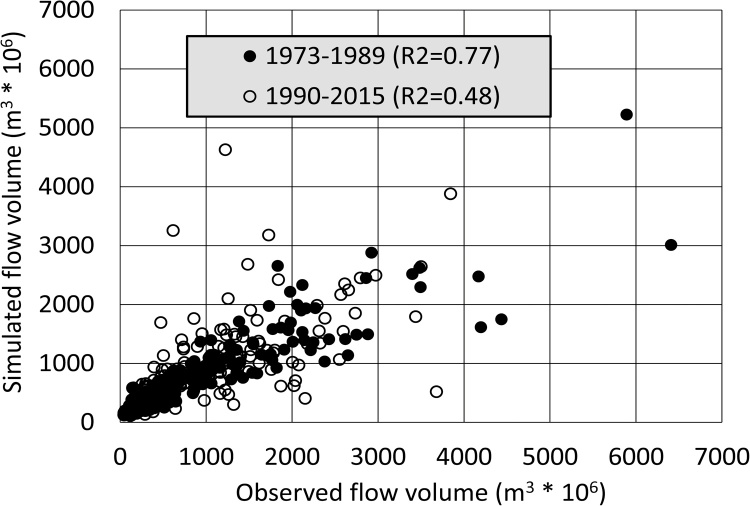
Scatterplots of observed versus simulated monthly flow volumes for Kafue Hook (KAF7) gauge (using WEAP model results) illustrating the possible effect of poorer rainfall estimates in the recent past.

The input climate data, and specifically the use of the CRU rainfall data, represent a large source of uncertainty in the model setups and results. This appears to be particularly true in the northern parts of the Lake Malawi sub-system, as well as over the Angolan parts of the basin. Previous experience with the CRU data in the Okavango River basin (Hughes et al., 2011b; Hughes and Gray, 2017) suggest that the rainfall data inputs to the western parts of the basin in Angola and parts of Zambia are particularly problematic, especially over the last few decades due to a reduction in the number of real rainfall observations. Fig. 8 also illustrates the same problem using the WEAP data for Kafue Hook (KAF7), where the earlier simulations (pre 1990) are better than the later ones (post 1990), particularly with respect to the simulations of the main wet season flows, a result that is partially attributed to the decreased representativeness of the rainfall data. However, some limited attempts to use alternative rainfall data that combine different sources of information (not relying solely on gauge data) did not improve the calibrations and some of these data sets only start in the late 1970′s (or later), which means that some of the earlier stream flow data (Table 2) could not be used in the calibration. While a comprehensive evaluation of many different data sets (Beck et al., 2017) could be useful, it would also be very time consuming, given that the models would almost certainly require re-calibration for each rainfall data set. However, from the perspective of simulating future water resources regimes, under a changing climate, it is essential that the main driver (i.e. rainfall) is estimated as well as possible. An evaluation of different estimation approaches, given the likelihood that the ground-based observation network is unlikely to improve, could therefore be critical for understanding future flow regimes and therefore water resources availability.

Understanding of the dynamics of water exchange between the rivers and the many wetlands in the system represents a further major source of uncertainty in several parts of the basin. The lack of detailed information about the existing water uses (groundwater, direct channel abstractions, storage and demand patterns, operating rules of hydro-power dams, etc.) in different parts of the basin, also impacts on the quality of the simulations. While some information is available (e.g. World Bank, 2010), it is recommended that a database of water uses should be established as a matter of some priority. Some of these data might need to be inferred from secondary information, such as land use patterns, but a great deal of the key data might be available from water use licensing information held by the relevant national water resources management authorities. Access to this information would therefore require a great deal of cooperation between the relevant authorities of the riparian countries and an appropriate study team. One of the key items of information that is required for running scenario analyses (Hughes and Farinosi, 2020) is realistic operating rules (hydro-power releases and flood control releases) for the major dams in the system.

While reducing some of the uncertainties referred to above will be beneficial, the overall conclusion is that the models have been established with sufficient confidence to suggest that they can be used for assessing future scenarios of both water use and management, as well as climate change.

## Software and data availability

The WEAP model and documentation is available from https://www.weap21.org/. The Pitman model is available as part of the SPATSIM modelling framework from https://www.ru.ac.za/iwr/research/spatsim/. Further details about the Pitman model are included in the documentation that can also be downloaded (see the Pitman_Guide.pptx file in the SPATSIM_V3/doc folder). The model setups (including the forcing data, parameter sets, simulation results, etc.) can be obtained on request from one of the authors, subject to some restrictions on the distribution of the observed streamflow data.

## Declaration of Competing Interest

The authors declare that they have no known competing financial interests or personal relationships that could have appeared to influence the work reported in this paper.

All authors have seen and approved the final version of the manuscript being submitted. They warrant that the article is the authors' original work, hasn't received prior publication and isn't under consideration for publication elsewhere.
